# Recommendations from recent graduates in medicine, nursing and pharmacy on improving interprofessional education in university programs: a qualitative study

**DOI:** 10.1186/1472-6920-14-52

**Published:** 2014-03-18

**Authors:** Conor Gilligan, Sue Outram, Tracy Levett-Jones

**Affiliations:** 1 University of Newcastle, Faculty of Health, School of Medicine and Public Health, Level 4 West HMRI Building, Callaghan, NSW, 2308, Australia; 2 University of Newcastle, Faculty of Health, School of Nursing and Midwifery, Richardson Wing, Callaghan Campus, Callaghan, NSW, 2308, Australia

## Abstract

**Background:**

Interprofessional education (IPE) has been recognized as an innovative approach for the development of a collaborative, practice-ready health workforce, but is not used consistently in undergraduate health professional programs. We sought to explore the reflections of graduates on the IPE experiences they had during their undergraduate education and training. It was anticipated that having completed their pre-vocational education and spent up to two years working in a clinical environment, recent graduates would be well-placed to provide insights into the value of the IPE opportunities they had, and to suggest approaches for improving these opportunities in undergraduate programs.

**Methods:**

This study was part of a larger research project (Interprofessional Education for the Quality use of Medicines; IPE for QuM) which used focus groups as part of an interpretive research design to inform other aspects of the research. Here, we report on focus groups with recent graduates recruited from area health services across Australia.

**Results:**

Sixty-eight recent graduates working in New South Wales, Western Australia, and Tasmania participated in 12 focus group sessions. In this paper, we report on new graduates’ reflections on their experiences of IPE as part of their university degree, as well as their recommendations to improve interprofessional education before graduation. The new graduates were unanimous in valuing IPE from their current perspective of being in the health workforce. Most IPE experiences recalled were regarded as positive, but those valued most highly were experiences that involved genuine engagement and opportunities to interact with students in other professions working on a relevant problem. Clinical placement was a missed opportunity with few structured meaningful interprofessional learning experiences. Surprisingly there was little social contact between professions in universities even when programs were co-located, thus reinforcing professional silos.

**Conclusions:**

The graduates provided many insightful reflections about the value of university-based IPE and their preparedness for clinical practice. Although universally acclaimed as a "good idea" there is much room for improvement. We put forward a set of suggestions to improve IPE and guide the design of future IPE efforts.

## Background

University-based health professional training largely occurs in discipline-specific silos. While this approach is appropriate for the teaching of many professional competencies, it is limited in its capacity to prepare students for the realities of working in interprofessional clinical teams. With 70% of adverse events in healthcare attributed to poor communication [1], it is imperative that adequate attention be given to the development of communication and teamwork skills in health professional education.

Interprofessional education (IPE) has been recognized as an innovative approach for the development of a collaborative, practice-ready health workforce [2]. "Interprofessional education occurs when two or more professions learn with, from and about each other to improve collaboration and the quality of care" [3]. The earliest IPE initiatives began in the UK in the 1960s, and a national movement was established in the 1980s [4]. Momentum for the IPE movement increased after the Bristol Royal Infirmary inquiry identified hierarchical teams and poor team functioning were associated with poor outcomes for babies undergoing heart surgery [5]. This led to a mandate that IPE be part of pre-registration training in all health and social care professions in the UK [5]. The Health Council of Canada also recommends that each university health sciences program offer an IPE subject [6]. In the United States in 2001, the Institute of Medicine published the report Crossing the Quality Chasm: A New Health System for the 21st Century which recommended that "health professionals should be educated to deliver patient centred care as members of an interdisciplinary team" [7].

In Australia, several government policy documents and independent reports have specifically advocated for the inclusion of IPE in health professional education programs [8,9]. The level of action on these recommendations, and evidence to guide IPE efforts are however, questionable, and there is as yet, no national IPE curriculum. A recent cross-sectional survey completed by 72% of all Australian and New Zealand Universities found that while 80% of universities claimed to offer IPE experiences to their students, the majority of these experiences did not fit the accepted definition of IPE (learning with, from and about other professions). Rather, the majority of the experiences involved passive learning from other professions in lectures or tutorials (29%). IPE opportunities on clinical placement accounted for 17% of the IPE efforts; however, most of these occurred without structured opportunities for interaction between students and staff from different health professions [10]. A 2005 literature review classified the various approaches to IPE, describing the levels as ‘bland’, ‘hat stand’ and ‘grand’ [11]. This model describes incremental benefits from each type of activity, but suggests that it is likely to be only the ‘grand’ approach (collaborative learning in the context f real clinical problems), which will break down attitudinal barriers between professions [11].

While support for the widespread implementation of IPE is strong and increasing, to date this trend has not been supported by rigorous research evidence. The most recent systematic review found only nine controlled trials of IPE, and reported that most evaluation focused on student attitudes towards, and perceptions of, IPE rather than actual learning outcomes [12]. Evaluation of educational efforts is inherently challenging and can limit the implementation of evidence-based learning opportunities.

One promising example of IPE in Australia is a six bed student training ward (STW) which operates within a 26-bed general medical ward at Royal Perth Hospital. The STW was established in 2010 and is the only current inter-professional training ward in Australia of which the authors are aware. Final year students from nursing, allied health and medicine undertake all ward duties as an inter-professional team and provide an inter-professional handover to afternoon shift staff. Facilitated group learning sessions and reflective practice complement profession specific and generic tasks that form part of student’s learning experiences. To date, only a very small proportion of Australia’s health professional graduates have been exposed to such experiences.

Given the lack of evidence for the short- or long-term learning outcomes from IPE experiences, we sought to explore the reflections of graduates on the IPE experiences they had during their undergraduate education. It was anticipated that having completed their pre-vocational education and spent up to two years working in a clinical environment, recent graduates would be well-placed to provide insights into the value of the IPE opportunities they had, and to suggest approaches for improving these opportunities in undergraduate programs.

## Methods

This study was part of a larger research project (Interprofessional Education for the Quality use of Medicines; IPE for QuM) which used focus groups as part of an interpretive research design to inform other aspects of the research.

Recent graduates working in the Hunter New England Area Health Service of New South Wales, Royal Perth Hospital in Western Australia, and Royal Hobart Hospital, in Tasmania were invited to participate in focus group sessions. Participants were recruited through posters displayed at each of the sites, as well as through graduate placement coordinators who sent invitations to eligible graduates working at each site.

Focus group discussions were semi-structured, using a series of open-ended questions designed to explore the participants’ recollections of IPE in their undergraduate programs, their sense of preparedness for clinical work, and their recommendations for improving IPE and clinical readiness. Discussions also explored participants’ experiences of working in interprofessional teams as part of their clinical roles.

The interview schedule was developed based on the aims of the larger project and the findings of the cross-sectional survey conducted as part of the project [10]. Each focus group was conducted by an experienced facilitator according to a prepared protocol. Each discussion lasted around one hour and was audio-recorded and transcribed.

Data was then analysed using iterative thematic analysis; one researcher identified a preliminary set of themes and sub-themes which were discussed and modified in an iterative process with two of the authors researchers (CG and SO). All data was then coded according to the agreed themes and sub-themes. This process is based on the method of thematic analysis presented by Malterud [13] and used effectively, more recently, by Frisch *et al.*[13,14].

Ethics approval was obtained from the relevant University and Health Services’ Human Research Ethics Committees.

### Participants

Sixty eight newly graduated health professionals from three Australian states participated in 12 focus groups. Thirteen health care participants were from Tasmania, 28 from NSW and 27 from Western Australia. Twenty-eight nursing graduates, 17 medical graduates and 23 pharmacy graduates participated. There were 8 discipline-specific groups and 4 inter-professional focus groups conducted. The focus groups varied in size from 2–10 participants per group. The eight focus groups conducted in NSW and Tasmania consisted of single professions. The four groups conducted in WA all contained nursing, pharmacy and medicine graduates and included seven (out of the total 25 participants) who had experienced the interprofessional STW at Royal Perth Hospital. These experienced are described elsewhere [15].

The mean age of the nursing graduates was 30.6 years old (range 21–54), medical graduates 23.9 (range 24–45) and pharmacy graduates 29.4 (range 21–39). 51 (75%) of the participants were female, 17 (25%) were male. These differences are broadly reflective of the demographic differences across university programs. Given the relatively small sample size and qualitative nature of the study, no statistical investigation of the demographic differences was performed.

## Results

Four areas emerged from the overall thematic analysis for the research: IPE at university; interprofessional communication experiences as new graduates; roles, responsibilities, and relationships; and patient wellbeing including medication safety and patient-centered care. In this paper, we will report only on the reflections of new graduates of their experiences of IPE as part of their university degree. The following themes arose from this data: experiences of IPE at University; missed opportunities on clinical placements; silos and social interaction; dissonance between stated faculty values and educational practice; and graduates’ recommendations to improve IPE. The findings relating to the other three areas are reported elsewhere [15].

### Experiences of IPE at university: the "bland approach"

Experiences of IPE during undergraduate education varied greatly, as would be expected with the participants having graduated from a range of universities and professional programs, none of which have a national curriculum. Participants discussed IPE experiences at university (lectures, tutorials and simulation labs); IPE on clinical placements; professional silos and wider university environment; and gave recommendations for IPE to improve graduate experiences.

The new graduates were unanimous in valuing IPE from their current perspective of being in the health care workforce. They articulated clear reasons why it was needed and that the knowledge and skills about working with other professions gained through IPE should be part of their pre vocational education.

*"…it would be really good to have some kind of class together, some kind of discussion together, particularly in their final year and in our final year, because in first year of medical school you’re not really going to have an understanding…But if they got that before they become an intern, and if we had that understanding before we graduate, I think that would really help consolidate things…"* (Female nursing graduate, TAS)

A small number of participants, while appreciating the importance of IPE, voiced doubts about whether this learning could be achieved outside of the workplace or clinical placement.

*"I don’t really remember being taught by other professions or being taught about other professions to a great degree. So unless you had pracs* [practical clinical placements] *in hospitals and out in the community, where you were given that extra opportunity, I don’t really see how people would get more of an understanding."* (Female nursing graduate, TAS)

Most students had common lectures with other students early in their program:

*"…huge lecture theatre filled with 140 students, 80 of us and 60 science students, so there was no real interaction between each other."* (Female pharmacy graduate, TAS)

The following excerpt not only describes such common learning experiences, but also implies a perceived hierarchy of professions, which is not broken down in common lectures and can contribute to poor interprofessional outcomes:

*"…We have shared lectures with dentistry students for the first two years, which is fine for us, but it’s more they’re sitting in on our lectures, it’s not really a shared learning…"* (Male medical graduate, NSW)

Messages given by lecturers about working with other professionals, although well meaning, were at risk of perpetuating negative stereotypes and having an adverse impact in the workplace. Another male medical graduate (NSW) articulated this in:

"…*you’re sort of playing mediator between the team and the nurses and trying to not get on the bad side of nurses, because you’ve always been told that’s a really bad idea*!"

Many graduates struggled to recall IPE experiences from their education program. Those who did mostly recalled didactic experiences such as common lectures. The experiences valued most highly were experiences that involved genuine engagement and opportunities to interact, rather than simply a lecture from another professional. Graduates were generally able to describe these interactive, practical experiences in far greater detail.

*"Yeah there was one day where they formed …us into teams that had medical students and pharmacy students, maybe pharmacy students…physio* [physiotherapy] *and OT* [occupational therapy] *and social work students, and nursing students, in each team. So there was one in each team, and that was good, that was good to sort of see how they approached the same problem…"* (Female medical graduate, NSW)

Other remembered and valued IPE experiences included a large tutorial of medical students with a pharmacist and paediatrician team-teaching about medication problems, students actually writing prescriptions and getting feedback, interprofessional simulations as part of ‘DETECT Between the Flags’ [16] training, and being taught practical clinical skills by other professionals.

### Clinical placements: missed opportunities

The most memorable IPE experiences seemed to occur in clinical placement settings, but if opportunities for interaction and learning on these placements were not structured, they were often ineffective or missed.

*"…when we were on our clinical rotations, we saw a couple of students, med students and nursing students…But there was no interaction…It would just happen to be that we were on rotations at the same time…we didn’t actually do any study or liaising with them about patients or anything like that, like it was very much segregated into "you are a med student, you are a pharmacy student." So, you don’t talk."* (pharmacy graduates, TAS)

It seems that many IPE experiences occur in an ad hoc fashion, and are not consistent for all students. They were largely limited to time constrained interactions and passive observation of each other’s roles.

*"…it wasn’t…part of our practical component to actually find and go and work alongside a physio or an OT."* (Male nursing graduate, WA)

Even some of the structured experiences were not available to all students.

*"I recall when I did my rehabilitation term…we had a speech pathologist come and presented to us …but I don’t think that was something for every person that did that rehab* [rehabilitative care] *term, I think that was something that was put together."* (Male medical graduate, NSW)

Importantly, the opportunity for more sustained contact such as within an elective, can transcend the more pragmatic learning about roles, to enable a deeper appreciation, leading to greater respect. A changed perspective is how one medical graduate remembers the impact of IPE on her.

*"…this year for my elective I did a four week* [block] *with the midwives, and being able to see things from their perspective changed my perspective of their job. Like I’m so much more respectful of what they do, because I’ve seen how they work."* (Female medical graduate, NSW)

Reinforcing the need for structure to guide student’s engagement with, and learning from, clinical placement experiences, participants saw limited value in experiences that consisted of observation such as being a passive observer at meetings.

*"…we had to go to multi-disciplinary team meetings on the odd occasion, as part of oncology. But I wouldn’t say they were helpful at all, and it was just so over our heads that it didn’t matter that there were other disciplines…there was nothing to gain from all those meetings we had to go to."* (Male medical graduate, NSW)

Had the students’ participation in these meetings been associated with a specific objective, question, or activity involving other meeting participants, the value is likely to have been far greater. This was further illustrated by a female medical graduate (NSW) in:

*"…as a student I just went to the allied health meetings of that ward, and just saw what the medical team was seeing, but that was pretty much it".* (Female medical graduate, NSW)

‘Seeing what the medical team was seeing’ might be regarded as a useful learning opportunity, but without some structured objective or conscious effort to explore or appreciate the perspective of other professions, learning opportunities may be missed. The statement sharply illustrates the unconscious professional lens through which students view clinical encounters, particularly in later years, and contrasts with the earlier comment by the student whose elective experience changed her "perspective".

Perhaps the most positive reflections relating to IPE in clinical placement were those associated with the student training ward in Perth. The feedback specific to this exposure is presented elsewhere [17], but briefly, participants perceived key benefits to be the opportunity to learn about the roles of other health professionals and feeling better prepared to ask questions when required. The pharmacy graduates also reflected on the value of learning about the administration of medications via routes other than the oral route, and medical students particularly valued the opportunity to practice writing medication charts.

### Silos and social interaction

A major underlying theme emerging from the focus group discussions was the existence of silos and the complexity of relationships between professions. Discussion of the tensions between professional roles and the pervasive stereotypes and attitudes which can shape interprofessional interactions are reported elsewhere [15], however it is interesting to note that students’ experiences at university rarely included attempts to break down the silos, thus potentially perpetuating the professional separation. Even social interaction between student groups was minimal.

*"In the library where we studied a lot of…I think nursing and physio* [physiotherapy] *and pharmacy students also studied, but we didn’t form study groups, or really interact very much."* (Female medical graduate, NSW)

Graduates also reflected upon the separate nature of the professional programs, with the wider implications for healthcare, beyond knowledge of roles clearly apparent.

*"… you sort of start to see Med* [Medicine] *as more exclusive…it’s different you know …I suppose there was that set, that really definite separation…"* (Female pharmacy graduate, TAS)

Participants did however, recognise the potential benefit of even informal social interaction in helping to break down some the preconceptions and stereotypes and provide some degree of ‘preparation’ for clinical teamwork upon graduating.

*"…it doesn’t have to be clinical. It can be something social, something fun, but you need it mixed and you need to be working towards some common goal … I think that promotes relationships and understanding of one another as well. I think that's more important than the actual um, knowledge of clinical situations, it’s more about building those relationships and that respect for one another."* (Female medical graduate, Perth)

### Dissonance between stated faculty values and educational practice

The concept of interprofessional teamwork and multidisciplinary care were familiar to all participants:

*"The multidisciplinary healthcare team comes up in every single lecture, really in clinical and in therapeutics…."* (Female Pharmacy graduate, TAS) and *multidisciplinary teams, that was the catchphrase of the whole degree I reckon …"* (Male pharmacy graduate, NSW)

Despite the stated importance of multidisciplinary healthcare and teamwork by faculty, it seems graduates did not feel well informed or prepared for what this meant in clinical practice.

*"I didn’t feel prepared as I hadn’t had any real scenarios…the problem had been highlighted, but there was no context."* (Female nursing graduate, WA)

*"…actually that’s one of the things that I’ve been reflecting on of late…how little to nothing I know about what physiotherapists …pharmacists…occupational therapists…social work does, and what they have to offer. Everything I know at the moment is purely through the experience of working…"* (Male medical graduate, NSW)

### Participants’ recommendations for future IPE

The participants made several useful suggestions for improving IPE. An overriding message was that a great deal of room for improvement exists, and thus even simple opportunities for interaction would be an improvement on the experiences of many graduates.

*"…anything … tutorial-wise, where you’re actually interacting and getting to know what the other people know, what their knowledge is, and…how you can help each other I think is going to help from currently, where yeah, we don’t really do anything at all."* (Male pharmacy graduate, TAS)

Graduates also emphasized the need to integrate IPE efforts within programs rather than including them as stand-alone activities.

*"it’s an important subject and it has merit, but it’s then got to be taught in amongst the other parts of the course…"* (Male medical graduate, NSW)

Several graduates emphasized the need to learn about the roles of others; something which could be done in the classroom prior to clinical placement to help guide learning experiences on placement.

*"You know even if it became one day, or one lecture, where they invited…the speech pathologists, the physiotherapists, the members of the multidisciplinary team to come and explain what their roles were in the hospital environment… if we could have a general overview of what…those people do… then we probably would’ve benefitted from that I think… earlier in your education rather than when you first start the job."* (Female Nursing graduate, NSW)

The details in graduates’ recommendations indicated unmet needs. They wanted not only to know the roles of other health professionals but also the limits of their knowledge.

*"… to have one of the lecturers that do teach the other professions come in and tell us ‘well you know, this is what we’ve taught them about medicines,’ or … ‘this is what nurses know overall about medications, and this is where the knowledge is going to end.’ And I guess to have that understanding when you graduate, so you’ve got an idea of how to talk to people about medications, or what you expect them to know, and what maybe you’ll have to go over and things like that."* (Male pharmacy graduate, TAS)

The graduates spoke not only of the functional aspects of knowing about other professionals’ roles to better utilize them in patient care, but of their hope that if they were better understood, then there might be greater respect.

*"…you’ve got to realise the work that goes into the other roles and things, and it’s not just you know, the doctor writing on the drug chart, there’s a lot more behind that…understanding that you do have a lot more respect for them and other people."* (Male pharmacy graduate, TAS)

*"I think a lot of doctors don’t actually have a clue what nurses do, to be honest. I mean they have orders, they get followed through, and as long as they get followed through, they don’t really notice what else we do. So they don’t know how we assess patients…they don’t trust our examinations."* (Female nursing graduate, TAS)

The nurses and pharmacists both talked extensively about the perceived lack of respect, which is explored in a separate paper.

Several suggestions focused on the use of simulation or role-play or case scenarios with other professions to learn about their roles, as well as teamwork skills:

*"It would be good to have case scenarios, with input from each of the different groups saying ‘this is what we’re looking at, and this is what we’re trying to figure out.’ And then you know, for us to say, ‘well these are the medications we’re worried about,’ or ‘this is what we’re looking at,’…and the nurses say, ‘well we’ve also got to look at this, this and this…"* (Female pharmacy graduate, TAS)

Many suggestions focused on maximising IPE opportunities while on placement by including structured learning activities. These activities would ideally focus on common issues or cases dealt with and relevant for a range of professions, such as medications, falls, stroke and discharge planning.

*"In the oncology rotation in Newcastle, and in the palliative care rotation, there is a… list of …optional tasks, of which you need to do two or three…amongst that is ‘spend the morning, spend the shift, or half a shift, with a member of the nursing staff, to shadow along and see what they do,’ … it’s not mandatory, and not everybody can do it, because there just simply aren’t enough nursing staff… I think, that certainly would help."* (Male medical graduate, NSW)

One interesting suggestion was the importance of having students of nursing, pharmacy, medicine working together, rather than for example a registered pharmacist working with the medical students. This perhaps indicates an awareness of the additional benefits of learning from each other, and building social relationships.

*"… in the placement, maybe medical students can do one day as a nursing student, maybe they can see how the nurses work…Just for one day, so they know what nurses are doing."* (Male nursing graduate, TAS)

The higher value and importance given by students to learning experiences on clinical placement, reinforced the need to focus on this area for IPE.

*"… as a competency [for] a student on placement…bringing that sort of stuff in as well (talking about medications, and communicating with other health professional students), because then you’re more inclined to do it on placement, as opposed to just flying past."* (Female nursing graduate, NSW)

The need for an increase in practical IPE experiences was captured by another participant:

*"…learning from the nurses when I did the student training ward, was fantastic…learned about the drugs, but the administration side was all very textbook…never get shown how to administer anything. So it’s all just reading what’s in a book basically....anywhere where they could integrate it into the university system…any kind of increase in inter-professional education at all would be fantastic you know, because we all have none from our degrees."* (Female pharmacy graduate, WA)

While it was emphasized that some IPE activities could occur at university and should begin early in each program, it was also acknowledged that as knowledge and clinical experience increase, so should the complexity of IPE activities increase.

The participants reflected on a lack of education relating to communication and a sense that they were unprepared for the interprofessional communication essential to their jobs. Some suggestions were made about how this might be improved, including a focus on interprofessional communication. Others included:

*"how to bring up you know, a possible error or something with a doctor, without undermining them."* (Female pharmacy graduate, TAS)

*"…is there an undergraduate education that prepares you to be brave enough and to communicate with other disciplines… …You never talk to doctors as a student."* (Female nursing graduate, NSW)

## Discussion

Many participants struggled to recall IPE experiences from their education program, reinforcing the limited nature of current IPE efforts in Australia. Our recent cross-sectional survey revealed that the majority (29%) of activities that were regarded as IPE in Australian and New Zealand universities were actually lectures or tutorials conducted by staff from other disciplines [10]. While such experiences may be successful in meeting objectives for content knowledge and theory, it seems that they have limited value in improving understanding or skills associated with interprofessional roles or team functioning. According to the classifiction system put forward by Sanson-Fisher et al. [11], most of the experiences described by participants in this study (common lectures) would be regarded as ‘bland’, being primarily didactic with no ‘active intellectual interaction’ between students. While such approaches provide a low-cost means of delivering large amounts of information to large groups of students, they are characterized by rigid delivery and minimal genuine interaction.

Most IPE experiences participants recalled were regarded as positive ones, but those valued most highly were experiences that involved genuine engagement and opportunities to interact, rather than simply a lecture from another professional. Approaches involving interprofessional groups of students working together in tutorial situations (but probably managed in a discipline-specific fashion) were classified as a ‘hat-stand’ approach in the review mentioned above [11].

Participants’ reflections and the themes identified were in keeping with the quantitative data collected from universities in our previous cross-sectional survey. While IPE opportunities on clinical placement accounted for 17% of the IPE efforts, most of these occurred without structured opportunities for interaction between students and staff from different health professions [10]. Without structure or guidance, students are likely to miss valuable opportunities for interprofessional learning and teamwork experience, particularly if pre-placement learning has not prepared them for the importance of interprofessional teams or provided knowledge about various professional roles.

Graduates’ comments about the need to integrate IPE efforts are in keeping with current efforts to use IPE approaches to teach about patient safety; identified as an area with common competencies for a wide range of health professions [18]. This is also highlighted by Sanson-Fisher et al. as one of the potential challenges of the ‘grand approach’ – finding places for common clinical competencies or learning objectives to be taught across disciplines can be challenging [13].

It would seem that practical experiences which are likely to represent the most beneficial learning experiences, with relevant, practical experiences allowing students to develop precise role definitions and practice collaborating constructively prior to entering the workplace. Such an approach represents the highest level of IPE classified by Sanson-Fisher et al. as ‘grand’ and while it offers potential benefits to students and patients, it is constrained by practical considerations such as timetabling, cost, and differences in accreditation requirements and competencies across disciplines [11].

Attitudinal change is a common objective of interprofessional education efforts but can be difficult to control and measure. ‘Tribalism’ afflicts many health professionals, and represents a rigid pattern which will not be changed in just one ‘generation’ of new graduates [19]. While ever students are being taught by professionals who hold ingrained professional stereotypes and attitudes, these attitudes will be perpetuated. Mezirow describes a key component of learning (particularly adult learning) as becoming ‘critically aware of the cultural and psychological assumptions that have influenced the way we see ourselves, our relationships, and the way we pattern our lives’ [20]. In overcoming many challenges, it is not enough to increase knowledge about the particular issue; rather, a greater transformation is required. This usually involves an understanding of the perspective of others and can result in attitudinal change. Such change can depend however on the ‘baseline’ attitude of an individual, as well as that of educators or facilitators with whom individuals have contact. Educators, clinical supervisors, and health professionals are in a prime position to guide students through a ‘transformation’ process, facilitating an adjustment in their lens and a capacity to view situations from another’s perspective.

Attempts to reform the education of health professionals to improve the quality of health care in recent decades has been extensive. However these changes have largely involved formal curricula. The pervasive influence of the hidden curriculum has been recognized as a potent force that may hinder the achievement of these reforms [21]. The hidden curriculum refers to lessons that are learned without being explicitly intended [21]. Poor interprofessional practice by, or a failure of, clinical placement supervisors and other educators to acknowledge interprofessional education opportunities is likely to teach students that this practice is not valued in the workplace. Greater attention needs to be paid to the wider learning environment including resource allocation, accreditation policies and importantly, positive role models for the way that patients, students and graduates are treated in the clinical environment [22]. The finding that multidisciplinary teams are mentioned a lot by lecturers, but that there was little effective IPE teaching and learning sends a powerful message via the hidden curriculum.

### Study limitations

This study used convenience sampling to recruit participants from a pool of recent graduates at the sites involved. While all recent graduates were invited, it is possible that participation bias was present with those graduates who took part being potentially those who had a greater interest in IPE. The representation from professional groups did not reflect the mix that would be present in the workplace. Also, the focus group questions directed discussion to cover key topics which may have limited the scope of discussion on other related issues. As such it is likely that additional issues not evident in our data will influence attitudes towards IPE and medication safety. Notwithstanding these limitations, the insights of the graduates involved provide a valuable snapshot of experiences from a range of universities at a range of clinical sites.

Our results are supported by the feedback arising from the student training ward in Perth, but this represents a unique opportunity, and one which is currently only available to a small number of students. Our findings and recommendations are also in keeping with the learning outcomes suggested by Thistlethwaite *et al.* in a recent review and synthesis of IPE programs [23], as well as the World Health Organisation’s patient safety curriculum [18]. The challenges of identifying the best approaches for implementing IPE and overcoming the pragmatic and cultural barriers however, still remain.

One graduate articulated a challenge that has been expressed by academics and clinicians alike in pursuing IPE efforts; that patient safety, interprofessional teamwork and interprofessional communication will only improve if a positive interprofessional culture exists in the clinical context. While-ever undergraduates who are taught best practice skills in communication, teamwork and interprofessional practice graduate and work in a climate of hierarchy, silos and stereotypes, any IPE efforts are destined to fail.

*" …I don’t know how…the university can change cultures within the health system. You can keep empowering your students and your undergraduates, and tell them that…’you’re within your rights, and it’s justified, and you need to do this’…but I suppose, maybe the undergraduates need to be educated that they’re going to confront roadblocks and cultures…in the hospitals."* (Nursing graduates, NSW)

This issue will require sustained effort not only in universities but in healthcare institutions and postgraduate training programs.

## Conclusion

The graduates participating in this study provided many insightful reflections about the value of university-based IPE and their preparedness for clinical practice. Taking into consideration the experiences of graduates in starting professional work, their reflections on university IPE experiences, and their recommendations for improving IPE, we have compiled the following set of suggestions to guide the design of future IPE efforts.

1. Begin early in the program with simple concepts about others’ roles and responsibilities and increase in detail and depth as programs progress and clinical situations become more relevant.

2. Encourage social interaction between students from various programs to promote familiarity for future work and placement interaction, and opportunities to share knowledge and experiences.

3. Each professional group should learn what the other professions know; what they are taught about particular topics and what their roles and skills are in particular situations.

4. IPE opportunities should be structured as part of clinical placement, associated with clear learning objectives.

5. Opportunities should be provided to work together on common clinical scenarios and problem-solving tasks, whether at university in classrooms and simulation labs, during clinical placement, or both.

6. Focus on communication tasks to develop the skills that are central to practice.

7. Address hidden curriculum through faculty development especially with clinical educators.

While these IPE initiatives can add value to a safe and effective workforce they will only reach their potential when parallel changes to improve the interprofessional culture occur in health care settings. This represents perhaps the greatest challenge for the future of IPE, and one which will require collaborative effort from the health workforce at individual, organizational, and structural levels.

## Competing interests

The authors declare that they have no competing interests.

## Authors’ contributions

CG and TLJ were involved in conceptualizing the study and preparing focus group protocol. SO and TLJ were involved with recruiting participants and facilitating focus groups. CG and SO conducted iterative thematic analysis and prepared the manuscript. All authors read and approved the final manuscript.

## Pre-publication history

The pre-publication history for this paper can be accessed here:

http://www.biomedcentral.com/1472-6920/14/52/prepub
